# Lecture on Muriatic Acid

**Published:** 1888-01

**Authors:** J. T. Kent

**Affiliations:** St. Louis


### LECTURE ON MURIATIC ACID.

PROFESSOR J. T. KENT., M. D., ST. LOUIS.

The object of this lecture is to show you how to individualize. The great failure in prescribing is not in securing a right potency, but in selecting the appropriate remedy. I may lead you year after year down through the symptoms of remedies, and you will not see how to grasp a group of remedies that seem quite generally similar, and detect their differences. We will take up to-day *Muriatic acid* and some remedies that seem somewhat like it, and see if we can generalize with them, and then individualize. There is no greater task after the symptoms have been duly appreciated than individualizing between two or more medicines that seem similar to the sickness to be cured.

The most prominent feature is muscular prostration, as a result of blood-poisoning. Muriatic acid occupies a prominent sphere in zymosis, zymotic diseases, typhoid, that peculiar state and condition of disease, marked by sepsis, associated with paralysis, weakness of the muscles of the body.

In a large number of the complaints there is a marked absence of any primary cerebral trouble, but later there comes on a marked cerebral condition, depending upon this zymotic state, cardiac paralysis. In the mind symptoms, we have loss of consciousness, coma, passive delirium, muttering, picking at the bed-clothes, tendency to slide down in the bed. During the coma there is moaning, always moaning; this state we find in typhoid fever; as we run down the typhoid fever it has a typical state, dryness, bleeding of the mucous membrane, exudation of dark blood, the tongue is coated; if the coat is removed it is bright red; or the tongue covered with dark brown or black coating; tongue is projected with difficulty.

Ulcers on the mucous membrane as well as on the skin. Ulcers with bleeding and burning; burning is a characteristic of Muriatic acid. Ulcers burn, eruptions burn, burning in the veins. It produces hemorrhoidal tumors, that protrude like grapes, burning when touched, like Phos. acid. Burning in the stomach, burning in the abdomen.

It produces crops of boils and carbuncles that burn and have sticking on the slightest touch. It produces an increased symotic condition of the skin, blue spots, like Arnica.

It sours the secretions of the body, acrid discharges, excoriate and burn, tendency to raw surfaces upon the body, exfoliation of the cuticle in patches.

In the abdomen we have extreme soreness, burning, bloating, tympanitic condition—drum-like; vomiting and diarrhœa, or diarrhœa without vomiting; vomiting of sour smelling water, vomiting of feces; sometimes profuse, gushing diarrhœa, with this there is great prostration; extreme offensiveness of the secretions and excretions, the breath is foul, and the patient smells badly; it corresponds to a low type of typhoid fever. There is something peculiar about the prostration as to its coming on; in some ways it would make you think of Phos. acid, but the Phos. acid patient becomes delirious, yet he is very strong, finally as a result of more cerebral congestion he takes on a weakness of the muscles; as a result of the brain congestion, the trouble starts in the brain. We will reverse that in Muriatic acid, the paralysis comes on from the sepsis, and finally the brain assumes its form of congestion and delirium. Muriatic acid is to the muscles what Phos. acid is to the brain, but if you see them in the advanced stage, they are both alike, they both slide down the bed, they both pick at the bed-clothes, in both unconsciousness, involuntary stools and urine, they both seem to enter into the extreme passive state, state of unconsciousness, that may be the result of typhoid or symptomatic typhoid.

He must strain and wait a long time for the urine to pass; finally, after straining a long time, the urine dribbles away. While pressing to urinate the rectum prolapses, he has prolapsus of the rectum, while pressing to urinate; you will not find that in any other remedy.

Now I have described as much of the typhoid state as Muriatic acid conforms to; there are a number of little symptoms in this remedy, but these are the principal ones. This remedy runs into association with many remedies that you will have to use in different climes and different diseases that sometimes assume the septic state, as measles, scarlet fever, and diphtheria have many conditions of typhoid sepsis and zymotic state; septic condition like puerperal fever, which takes on a rapid typhoid, and we have a large number of remedies running in. Now let us draw an imaginary circle. And now let us put inside of that circle the pathognomatic symptoms of typhoid fever. A typical case of typhoid comes on slowly. The patient feels badly for weeks before he is confined to his bed. The diarrhœa finally comes on with offensive mushy stools, yellow or brown, and there is increasing prostration and the fever is continued. Read up the books and gather the symptoms there named as belonging to the continued or typhoid fever; take them bodily and write them inside of this circle. No matter what name you apply to this circle or complexity of symptoms. This you must do at every typhoid bedside. At a glance you will learn to see a general similarity standing out of this group that will remind you of the picture or image of some remedies used for its cure. Related to the drug in mind, we have especially Arn., Ars., Bry., Bapt., Phos., Phos-ac., and others that might confuse you to mention. We must first see the similarity in the images considered generally, and then we must take up the list and see why they are not all in perfect consonance with the symptoms of the patient. They all may have in a greater or less degree the pathognomonic symptoms in the circle, and yet not one of them be able to cure the sickness. Generally, they may all be similar but specifically quite dissimilar. Thus far we have only stated that there is a general similarity between these remedies and Muriatic acid, and only a general similarity between Muriatic acid and the group of symptoms in the circle. In relation to the specific study of these medicines there must be some objective point, hence we have taken the fragmentary image in the circle. We have a marked typhoid image in Muriatic acid, so we have in all the rest of the named medicines. When shall we give one and when shall we give another. Every case you meet will furnish you a new problem.

Suppose we start with Arnica. It produces great muscular weakness, and the unconsciousness like Mur.-ac.. but the Arn. patient comes down more rapidly; he becomes stupid rapidly and says he has been bruised; he is so sore all over and complains of the hardness of the bed. He must not be touched. We do not find this soreness in Muriatic acid. Both have ecchymoses, bluish spots, brown sordes on the teeth, and bloody exudations. In Arnica he forgets the word while speaking. He attempts to say something in answer to your interrogations and fails because he has forgotten the words to express his ideas; he becomes provoked at this and drops into a stupor. If again aroused he becomes very irritable and declares he is not sick, that he did not send for a doctor, and does not need a doctor. He will do all this when he is distressingly and dangerously sick. He may refuse to take the medicine, and then swallow it without resistance. He has offensive, gushing, inky stools, that come on even in the first days of his sickness. These symptoms coming prematurely in typhoid fever suddenly, the activity of the symptoms is peculiar. If these symptoms should come on later one could scarcely think of Arnica—I mean the diarrhœa symptoms. In Muriatic acid the brain symptoms come on later, and also the diarrhœa.

In Arsenicum we see much of the image that is similar to Mur.-ac. The extreme prostration is a grand symptom of Arsenicum. It seems that he will die; he can hardly move a hand, he is so debilitated, so weak. Perhaps he has passed through a period of restlessness and extreme mental anguish in which he finds no rest. He goes from chair to bed and from one bed to another.

Moaning all the time, finding no comfort. Even the anguish is depicted on his countenance. He has a constant fear of death. He is thirsty, and the most typical feature of his thirst is that he wants just water enough to moisten his mouth and throat; thirst for large quantities frequently is a strong feature in Arsenicum. Craving for hot drinks is often found. Frequent, scanty, bloody, brown, black, cadaverous stools. In Muriatic acid the stools are copious.

Arum.-tr. covers typhoid states, sepsis, blood-poisoning, etc. The foul mouth, raw and bleeding lips, tingling of nose, compelling him to bore the nose with the fingers, which is already raw and bleeding. He picks the tips of his fingers until they become sore, also the lips and nose; urine is scanty or suppressed. He passes into unconsciousness and slides down in bed. Mushy stools very frequently day and night.

Baptisia has a marked general zymotic state. So true is this that unskilled homœopaths will say, "Baptisia for typhoid fever," without pointing out when it is indicated and when it is inappropriate. The homœopathist will always say, *When shall I prescribe it?* The patient has a besotted expression, as if he had been on a debauch with strong drink. He looks as if his condition had come on slowly, but it has come on suddenly. The stupid condition has been but a few days coming to coma. He will at first answer your question and drop into a stupor. Like Muriatic-ac., his jaw drops and there are bluish spots on his face; watery, offensive stool associated with this typhoid state; the stool is so extremely offensive that you can smell it all over the house. It is death-like, it permeates the house, you can carry it with your clothing. We have extreme offensiveness in Bapt.; it has a little restlessness at times but he will draw up his knees and lie over on one side, and lie there for days and will not speak to anybody; he attempts to answer and falls asleep; if he has wandering, as we sometimes find him, he seems to be scattered all over the bed, and seems to want to get the limbs together; he thinks he is made up of numerous factors; he thinks his limbs are talking to each other and it annoys him; he wants to get them together.

The Bryonia patient is full of pains and aches, sore and bruised, diarrhœa, irritable in the extreme, and is only contented when let alone and permitted to lie quiet, and not urged to move. Bry. has the pains and aches that belong to the typhoid state, all made worse from motion; it has the mushy diarrhœa, the tympanitic abdomen, brown tongue, sordes on the teeth, great prostration, complaints come on slowly and the fever is continued; Bry. has these things. Now, in short, that is how Bry. competes with Muriatic acid, which has a desire to move, and better from motion, while Bry. is worse from motion.

Carbo veg. enters into this state; it has the prostration, the tympanitic abdomen, the pathognomonic symptoms of the typhoid state; it is horribly offensive, the breath and feces, and particularly is he filled with gas, which he passes, and it is very offensive; he can hardly tolerate himself; he has a sickly appearance; he seems sinking; the nose and the expression of the face are cadaverous; he wants air, he wants the windows open, and wants to be fanned; wants some one on either side of him to fan him, which gives him relief.

China has it all too; has the whole typhoid fever; while it is more characteristic for China to have periodicity, yet there are times it has a continued fever. It is indicated for the deathly sinking belonging to the last stages of typhoid, the watery, involuntary stools, profuse, painless, watery diarrhœa, hemorrhages or after hemorrhages; when the diarrhœa gives you the key-note, remember this, that where the bowels don't move in the day except after nourishment, and where the diarrhœa is frequent at night, of dark, inky fluid, don't fail to give China; exudations about the teeth, bleeding mucous membranes, blood-poisonings.

Colchicum compares with Muriatic acid, the aching pains, the muscular soreness, the swelling of the joints, the watery diarrhœa and the stomach disorder, that is peculiar to Colch., the thought or smell of food, the smell of cooking food, the mere mention of oysters, soup, or broth will make the patient gag; sickness, nausea, horrible aversion to something to eat; so they go on for days and days, until you cure it; there are lots of cases lost for the want of knowledge of Colchicum; I have known patients to actually vomit and retch when hearing some one speak of something to eat. You need not wait long for it to help that condition. Cocculus is something like it, but not compared here.

Gels. has the muscular prostration of Muriatic acid; it is expressed in Gels. and not expressed in Muriatic acid. You have to see it; he does not realize that he is so weak, but Gels. will tell you "I am so tired, always so tired, and my limbs feel so heavy." He has the mental power to understand it and express it to you; in cardiac trouble he will tell you about it.

If you will prescribe on these indications you will never have a typoid fever, because it will not get there.

Any prescriber ought to stop a common case of typhoid fever in ten days, allowing two or three days in the start without medicine.

